# Relationships between Prenatal Distress and Infant Body Mass Index in the First Year of Life in a Lower-Middle Income Country

**DOI:** 10.3390/ijerph17197351

**Published:** 2020-10-08

**Authors:** Ann-Sophie Therrien, Giovanna Buffa, Amanda B. Roome, Elizabeth Standard, Alysa Pomer, Jimmy Obed, George Taleo, Len Tarivonda, Chim W. Chan, Akira Kaneko, Kathryn M. Olszowy, Kelsey N. Dancause

**Affiliations:** 1 Department of Physical Activity Sciences, University of Quebec in Montreal, Montreal, QC H2X 1Y4, Canada; ann-so.therrien@hotmail.com; 2 Department of Anthropology, Binghamton University, Binghamton, NY 13902, USA; giavanabuffa@gmail.com (G.B.); estanda1@binghamton.edu (E.S.); 3 Bassett Research Institute, Mary Imogene Bassett Hospital, Cooperstown, NY 13326, USA; Amanda.Roome@bassett.org; 4 Department of Chronic Disease Epidemiology, Yale School of Public Health, New Haven, CT 06520, USA; alysa.pomer@yale.edu; 5 Ministry of Health, Port Vila PMB 9009, Vanuatu; manaruru@gmail.com (J.O.); gtaleo@vanuatu.gov.vu (G.T.); ltarivonda@vanuatu.gov.vu (L.T.); 6 Department of Parasitology, Graduate School of Medicine, Osaka City University, Osaka 545-8585, Japan; aramidus44@gmail.com (C.W.C.); akira.kaneko@ki.se (A.K.); 7 Island Malaria Group, Department of Microbiology, Tumor and Cell Biology, Karolinska Institutet, 171 65 Stockholm, Sweden; 8 Department of Anthropology, New Mexico State University, Las Cruces, NM 88003, USA; kolszowy@nmsu.edu

**Keywords:** mental health, maternal and child health, body composition, obesity, reproductive, maternal, newborn, child, and adolescent health (RMNCAH), developmental origins of health and disease (DOHaD)

## Abstract

Prenatal stress affects body composition in childhood and later in life. However, few studies assess body composition in infancy. Furthermore, most are in high-income countries and do not consider interactive or curvilinear relationships. We assessed distress and diet during pregnancy via questionnaires among 310 women in Vanuatu, a lower-middle income country. We measured body mass index (BMI) among 54 infants at 4–12 months of age. We analyzed interactive relationships between prenatal distress and diet with BMI Z-scores, and curvilinear relationships between distress and BMI Z-scores. There were no direct linear or interactive relationships between prenatal distress or diet with BMI Z-scores. We observed curvilinear relationships between prenatal distress and BMI Z-scores (*p* = 0.008), explaining 13.3 percent of unique variance. Results highlight that relationships between prenatal stress and body composition are evident in infancy but might not be detected if only linear relationships are assessed. Analyses in more diverse samples might help to explain inconsistencies in past studies.

## 1. Introduction

Research in the developmental origins of health and disease demonstrates that a stressful prenatal environment has long-term implications for physical growth and cardiometabolic outcomes such as obesity and diabetes. This likely reflects the effects of maternal stress hormones on developing fetal systems and epigenetic changes in fetal tissues that might have lifelong effects [1,2]. Prenatal stress exposure might affect the developing hypothalamic pituitary adrenal axis and thereby affect growth and cardiometabolic outcomes across childhood, adolescence, and adulthood [1]. Furthermore, prenatal stress is associated with low birth weight [3], which is a risk factor for obesity and could mediate or exacerbate relationships between prenatal stress and body composition.

Several gaps remain in the literature on prenatal stress and physical growth, body composition, or obesity. First, few studies assess relationships in the first years of life, although available studies indicate that relationships might be evident even in early childhood. For example, studies in the U.S. indicated smaller body mass index (BMI) Z-scores but greater central adiposity at age 3 in association with maternal corticotropin-releasing hormones, a biomarker of stress, during pregnancy [4]. Studies in Canada showed that stress due to natural disaster exposure in early pregnancy predicted increased adiposity at age 2 and a half and a greater increase in BMI Z-scores from ages 2 and a half to 4 [5]. More studies in the first years of life are necessary.

Second, most studies on prenatal stress and physical growth, body composition, or obesity are from high-income countries [2]. Results might differ in low- and middle-income countries (LMICs). For example, studies in Brazil (upper-middle income) showed that greater perceived stress during pregnancy predicted lower BMI Z-scores at ages 5–8 [6]. This is in contrast to many studies from high-income countries, which tend to show increased obesity risk following prenatal stress exposure [2]. More detailed studies in LMICs might highlight pathways to improve maternal and child health in these settings.

Finally, past studies tend to assess direct linear relationships between prenatal stress and physical growth, body composition, or obesity. However, they also point to complexities that warrant further study. For example, prenatal stress likely interacts with prenatal diet [1,7], but few studies combine analyses of these factors [2,7]. Furthermore, some studies indicate non-linear relationships between prenatal stress and child outcomes [8,9], but most focus on adverse effects of prenatal stress, with little discussion of potential curvilinear relationships. Research on these nuanced relationships in more diverse samples might shed light on patterns underlying conflicting results and point to new areas of study.

### ‘’Healthy Mothers, Healthy Communities” Study

In 2015, we created the “Healthy mothers, healthy communities” study in Vanuatu, a lower-middle income country in the South Pacific, to assess the role of maternal stress and diet during pregnancy on infant development. Our objective in the current study was to assess relationships between distress and diet during pregnancy and infant body composition, via BMI Z-scores, in the first year of life. We aimed to assess interactive relationships between distress and diet on infant BMI and curvilinear relationships between distress and infant BMI.

## 2. Materials and Methods

This study was approved by the Institutional Committee on Ethics for Research Involving Humans at the Université du Québec à Montréal, and the Vanuatu Ministry of Health.

### 2.1. Sample

In June–July 2016, we distributed questionnaires regarding mental health and diet among women of reproductive age in Vanuatu. Recruitment was through convenience sampling, primarily at Vila Central Hospital, which provides prenatal care for women across the archipelago. Following delivery, we analyzed available birth records to assess relationships between prenatal distress and diet with birth outcomes. We identified birth records for 310 women who completed questionnaires during pregnancy. Descriptions of the original sample and analyses of birth outcomes are detailed elsewhere [10].

In June–July 2017, we completed a follow-up among this cohort. Longitudinal studies in Vanuatu are complicated due to the distribution of the population across 68 islands and lack of detailed medical records, formal addresses, and telephone or email for many families. We identified 56 mother-infant dyads from the original cohort with complete data on prenatal distress. These dyads participated in assessments of maternal distress postpartum and infant body composition. Infants ranged from 4–12 months of age at the time of the follow-up.

### 2.2. Prenatal Assessments

Prenatal data collection was via questionnaires in Bislama, a Melanesian pidgin language used across Vanuatu. The development and properties of the distress scale are detailed elsewhere [10]. Briefly, we translated the Kessler-10 Distress Scale [11] and the Center of Epidemiological Studies Depression Scale [12] into Bislama and reviewed them with native speakers. After removing redundant questions, our final questionnaire consisted of 15 items assessing symptoms of anxiety, depression, nervousness, and stress—referred to here as “distress”—over the previous week. Responses were on a 4-point scale from 1 (not at all) to 4 (all the time). The mean score was used in analyses. Diet was assessed via 24-hour recall and a food frequency questionnaire. We classified foods into nine groups based on micronutrients using the Women’s Dietary Diversity Score [13], a widely used indicator of micronutrient adequacy. Dietary diversity was calculated as the number of groups represented in the 24-hour recall.

### 2.3. Postpartum Assessments

Women completed the same distress questionnaire during postpartum assessments. We measured infant length using a portable measuring board. To assess infant weight, we weighed mothers alone and then while holding their infant and calculated the difference. We computed sex- and age-specific BMI Z-scores based on World Health Organization standards [14]. BMI Z-scores were chosen to facilitate comparison with other studies.

## 3. Analyses

We examined descriptive statistics and correlations among variables. Given the small sample size, we first tested interactive relationships between distress and dietary diversity on BMI Z-scores, and curvilinear relationships between distress and BMI Z-scores, including only infant age as a covariate. In Model 1 (interactive relationship), we entered infant age (Block 1), followed by dietary diversity (Block 2), distress (Block 3), and the interaction between dietary diversity and distress (Block 4). In Model 2 (curvilinear relationship), we entered infant age (Block 1), followed by distress (Block 2), and the quadratic term for distress (Block 3). To validate significant relationships, in Model 3, we added other covariates in Block 1, including infant sex, birth weight, maternal postpartum distress, and dietary diversity during pregnancy. Analyses were conducted with SPSS version 22.0 (IBM Corp., Armonk NY, USA). *p*-values less than 0.05 were considered statistically significant.

## 4. Results

### Descriptive Statistics

Of 56 mother-infant dyads who completed the follow-up evaluations in 2017, two were excluded from analyses because they lacked data on infant BMI. Final analyses included 54 mother-infant pairs with complete data on prenatal diet and distress and infant BMI Z-scores.

Key characteristics of the 54 women did not differ from other participants in the original sample of 310 women. Mean scores for distress were similar in the follow-up sample of 54 (2.0, SD = 0.5) and those who did not complete the follow-up (1.9, SD = 0.5) (*p* = 0.416), as were mean scores for dietary diversity during pregnancy (4.1, SD = 1.3; 4.0, SD = 1.3; *p* = 0.438) and infant birth weight (3.351 kg, SD = 0.501; 3.224 kg, SD = 0.498; *p* = 0.171).

Among the infants, 35 (64.8 percent) were boys and 19 (35.2 percent) were girls. Mean age at time of follow-up was 9.4 months (SD = 1.6), and mean BMI Z-score was 0.50 (SD = 1.35). The mean maternal postpartum distress score was 2.0 (SD = 0.4). BMI Z-scores were not correlated with prenatal dietary diversity (r = −0.212, *p* = 0.132) or distress (r = −0.030, *p* = 0.830).

**Figure 1:**
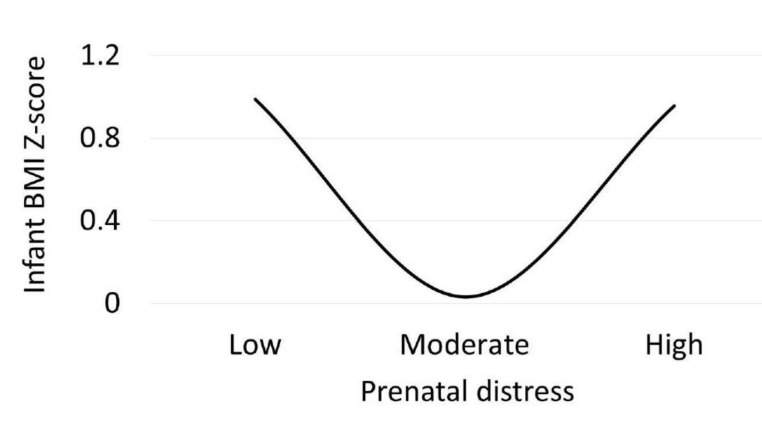
Relationship between prenatal distress and infant body mass index (BMI) Z-scores: Predicted values for BMI Z-scores plotted from results of regression analyses. Values for low, moderate, and high distress represent the 10th, 50th, and 90th percentiles in the current sample.

Table 1 shows the results of the regression analyses. Model 1 showed no interactive relationships between prenatal dietary diversity and distress with BMI Z-scores. Model 2 indicated a curvilinear relationship between prenatal distress and BMI Z-scores (*p* = 0.008), explaining 13.3 percent of unique variance (Figure 1). In Model 3, we validated the curvilinear relationship by controlling for key covariates in Block 1. There were no significant relationships between BMI Z-scores and infant sex, birth weight, age, prenatal dietary diversity, or postpartum distress. The curvilinear relationship between prenatal distress and BMI Z-scores remained significant (*p* = 0.034), explaining 8.3 percent of unique variance. On the other hand, given the large number of variables for the sample size, the model itself showed only a trend toward statistical significance (*p* = 0.080).

## 5. Discussion

Our objective was to assess relationships between prenatal distress and infant BMI Z-scores in the first year of life in a lower-middle income country. Although a number of studies in LMICs have assessed relationships between prenatal stress and physical outcomes at birth, such as birth weight, few have assessed longer-term effects of prenatal stress on child growth and development [15]. Our results highlight no direct relationships between prenatal distress and BMI Z-scores, nor interactions with prenatal diet, but a curvilinear relationship between prenatal distress and BMI Z-scores. Results indicate a higher BMI for infants with both low and high prenatal distress exposure. This curvilinear relationship remains evident when controlling for key covariates, such as infant birth weight, prenatal diet, and postpartum distress, although the model including all covariates must be interpreted cautiously given the limited statistical power. Results demonstrate that relationships between prenatal distress and BMI are evident even in infancy and highlight the importance of assessing non-linear relationships. Higher BMI in infancy might be considered an adaptive response to prenatal stress and might be beneficial in LMICs where childhood underweight remains prevalent. On the other hand, given that high BMI in the early months of life is a predictor of childhood obesity [16], these early growth patterns might have long-term public health consequences where the prevalence of childhood obesity is already high or increasing.

Most past studies on prenatal stress and physical growth, body composition, or obesity represent high-income countries. These tend to show increased obesity risk in childhood and adolescence following prenatal stress exposure [2]. For example, studies in Denmark showed that higher salivary cortisol during pregnancy predicted overweight at ages 2–16 [17]. Studies in Canada showed that prenatal stress due to a natural disaster predicted obesity risk at age 5 and a half [18], and relationships between prenatal stress and BMI became more pronounced with age [19]. Similarly, Danish National Register studies indicated that bereavement during or shortly before pregnancy predicted overweight in adolescence [20].

However, results are not always consistent. Studies in Denmark showed no associations between prenatal distress at 30 weeks of pregnancy and overweight at age 7 [21]. Similarly, the Amsterdam Born Children and their Development study showed that maternal job strain during pregnancy did not predict body composition at age 5. Furthermore, maternal cortisol showed only marginal relationships with fat mass index, with positive relationships among girls and negative relationships among boys [22]. Retrospective studies in Poland showed that maternal stress predicted an increased risk of underweight among boys at ages 7–10 and decreased risk among girls, although relationships with overweight were not assessed [23]. Finally, studies from Brazil—one of the few LMICs represented—showed that contrary to results from most high-income countries, prenatal stress predicted smaller BMI *Z*-scores at ages 5–8 [6].

Results from the current study might help to shed light on these inconsistencies. Most past studies have analyzed direct linear relationships between prenatal stress and body composition or obesity risk. However, relationships between prenatal stress and developmental outcomes are complex and depend on the characteristics of the stressor, as well as the capacity to respond. Chronic or high levels of stress might overwhelm the physiological response capacity and thereby have adverse consequences for offspring development. On the other hand, exposure to some level of maternal stress hormones is necessary and adaptive for fetal development [24], although few studies assess these relationships. Studies in the U.S. showed that greater prenatal stress predicted enhanced motor development at age 2 [8], and studies in Canada showed that moderate prenatal stress predicted better childhood cognitive development at age 5 and a half [9]. Our results suggest, similarly, that prenatal stress might have curvilinear relationships with BMI, with both low and high levels of stress promoting larger BMI. Thus, relationships between prenatal stress and BMI might not be evident if only linear relationships are assessed. Furthermore, it might be possible to observe contrasting relationships among samples based on differences in overall stress levels. Among samples in which stress levels or exposure are modest, we might expect negative relationships with BMI, whereas relationships might be positive in samples exposed to severe stress.

Differences in maternal response to chronic and acute stress might also underlie inconsistent relationships [3]. Systematic reviews show that direct positive associations between prenatal stress and body composition or obesity are usually observed in studies of natural disasters, which might represent more acute stress [2]. Results are less consistent in studies of perceived stress, longer-term life events, and other chronic stressors. We observed such differences in analyses of prenatal stress and birth outcomes in Vanuatu. Higher maternal distress due to a cyclone in 2015 showed direct negative relationships with birth weight [25]. However, relationships between chronic stress and birth weight among women sampled in 2016—the cohort from which the current sample was drawn—were evident only in interaction with maternal diet [10]. Relationships with later body composition measures might also differ. In cases of acute stress, we might expect direct positive relationships, whereas in cases of chronic stress, relationships might be curvilinear or evident only in interaction with other prenatal characteristics. Overall, these results highlight the importance of assessing interactive and curvilinear relationships in studies of perceived stress or chronic stressors and infant development.

### Strengths and Limitations

These results must be interpreted cautiously given the small sample size. In particular, statistical power to detect interactive effects is limited, and the lack of an interactive effect between prenatal stress and diet must thus be re-tested in other samples. Furthermore, our questionnaire measure cannot be used to diagnose mental illness or to distinguish between stress, anxiety, and depression, which might have different relationships with infant outcomes. Finally, data were collected at only one point during pregnancy and postpartum, which provides a limited perspective of the fetal and infant environment. More detailed assessments, including multiple measures across pregnancy and following delivery, would provide a more nuanced perspective.

Despite these limitations, our study is one of only a few assessing relationships between prenatal stress and body composition in infancy, and one of only a few representing LMICs. Results are strengthened by the prospective data collection on prenatal distress, such that women’s responses were not biased by infant characteristics. Furthermore, our questionnaires were developed from commonly used tools and included multiple questions to provide a more precise perspective of maternal mental health than in many other studies in LMICs.

## 6. Conclusions

Links between prenatal stress and body composition are well documented, but inconsistencies remain. Analyses in more diverse samples and consideration of non-linear relationships might help to clarify these complexities. Ultimately, such studies could guide efforts for early identification of infants at risk of adverse developmental outcomes following prenatal stress exposure.

## Figures and Tables

**Table 1:** Summary of regression models testing predictors of body mass index (BMI)-Z scores. Beta = standardized regression coefficient. Significant values in bold.

	Model 1 (F = 1.402, *p* = 0.248, R^2^ = 0.107)			Model 2 (F = 2.993, *p* = 0.040, R^2^ = 0.158)			Model 3 (F = 1.997, *p* = 0.080, R^2^ = 0.239)		
	Beta	*p*-Value	Unique R^2^	Beta	*p*-Value	Unique R^2^	Beta	*p*-Value	Unique R^2^
**Covariates**									
Sex (boy = 0, girl = 1)	-	-	-	-	-	-	0.01	0.957	0.123 ^a^
Birthweight	-	-	-	-	-	-	0.19	0.151	
Distress postpartum	-	-	-	-	-	-	−0.20	0.162	
Infant age (months)	−0.11	0.441	0.023	−0.18	0.191	0.023	−0.17	0.195	
**Pregnancy characteristics**									
Dietary diversity	0.062	0.302	0.041	-	-	-	−0.10	0.457	0.033
Distress	0.54	0.227	0.003	**−2.90**	**0.008**	**0.001**	**−2.35**	**0.038**	**0.001**
Diet * Distress (Interaction)	−0.98	0.158	0.039	-	-	-	-	-	-
Distress squared (Curvilinear)	-	-	-	**2.89**	**0.008**	**0.133**	**2.39**	**0.034**	**0.083**

(a) R^2^ for Block 1, all covariates.
